# Performance boost for primary magnesium cells using iron complexing agents as electrolyte additives

**DOI:** 10.1038/s41598-018-25789-8

**Published:** 2018-05-15

**Authors:** Daniel Höche, Sviatlana V. Lamaka, Bahram Vaghefinazari, Tobias Braun, Rokas P. Petrauskas, Maximilian Fichtner, Mikhail L. Zheludkevich

**Affiliations:** 1 Helmholtz-Zentrum Geesthacht (HZG), MagIC-Magnesium Innovation Centre, Max-Planck Str. 1, 21502 Geesthacht, Germany; 2 Helmut-Schmidt-University University of the Federal Armed Forces, Faculty of Mechanical Engineering, Holstenhofweg 85, 22043 Hamburg, Germany; 3 Helmholtz Institute Ulm (HIU), Helmholtzstr. 11, 89081 Ulm, Germany; 4 University of Vilnius, Department of Inorganic Chemistry, 03225 Vilnius, Lithuania; 5 Karlsruhe Institute of Technology (KIT), Institute of Nanotechnology, Hermann-von-Helmholtz Platz 1, 76344 Eggenstein-Leopoldshafen, Germany; 6 University of Kiel, Faculty of Engineering, Kaiserstrasse 2, 24143 Kiel, Germany

## Abstract

Aqueous Mg battery technology holds significant appeal, owing to the availability of raw materials, high power densities and the possibility of fast mechanical recharge. However, Mg batteries have so far been prone to decreased capacity due to self-corrosion of the anodes from the electrochemical redeposition of impurities, such as Fe, which results in parasitic cathodically active sites on the discharging anode. This work demonstrates that by adding Fe^3+^-complexing agents like Tiron or salicylate to the aqueous electrolyte of an Mg battery, it was possible to prevent the redeposition of Fe impurities and subsequent self-corrosion of the anode surface, thereby boosting battery performance. To prevent detrimental fouling of anode surface by Mg(OH)_2_, employed Fe^3+^-complexing agents must also form soluble complexes with Mg^2+^ of moderate stability. The interplay of these requirements predetermines the improvement of operating voltage and utilization efficiency.

## Introduction

Aqueous Mg-air batteries possess numerous appealing qualities for energy storage, including high volumetric capacities of metallic Mg anodes (3832 mA h cm^–3^, vs. 2061 mA h cm^–3^ for Li)^1^. Moreover, they use raw materials that are low in cost and relatively environmentally benign^2–6^ - indeed, such batteries for the first time can efficiently work even with ubiquitous electrolytes such as seawater^7^. Although aqueous Mg batteries are not electrochemically rechargeable, the option for fast mechanical recharging^8^ allows this technology to have numerous applications. For example, pilot projects for powering cars, have been accomplished at the Korea Institute of Technology in Seoul^9^. But why they are not available in large-scale on the market today? It is interesting that already in 1943 water-activated silver chloride/Mg-battery was commercially accessible^10^ however, it felt out of favour due to its low efficiency compared with nickel-metal hybrid and lithium batteries. And even 75 years later, a breakthrough in working efficiency for Mg primary systems has yet to be achieved under real-life conditions, regardless of whether the cathode is air or silver chloride. The novel concept introduced here might be the key.

In addition to obtaining suitable anode^11^ and cathode materials^12,13^, the electrolyte itself is a challenging component of any type of Mg battery^13,14^. So far economically attractive aqueous electrolytes cause problems related to the self-corrosion of Mg anodes^8^. First, the electrochemical potential of Mg is highly negative, and lies lower than the electrochemical stability window of water, thus causing its reduction and self-corrosion of the Mg anode. In contrast, the kinetics of water reduction on a pure Mg surface covered with an oxide film are rather slow, thus resulting in a lower extent of self-corrosion.

Second, Mg is also prone to corrosion when accompanied by noble impurities such as Fe, Cu or Ni^15^. Fe-rich particles, present in commercial magnesium, are particularly critical, because they allow for high exchange current densities in the hydrogen evolution reaction (HER) and cause highly localized microgalvanically induced corrosion of Mg^15,16^, thereby triggering the growth of corrosion products on the surface of anodes that block the electrodes^17^. They consist of a very thin layer of MgO directly at the metallic interface, gradated porous hydroxide on top and partially carbonates. Latter can be a mixture of MgCO_3_ xH_2_O, or mixtures with - (OH)_2_ depending on pH, solubility product constant and concentration of carbonyl groups.

The self-corrosion of Mg anodes through these two phenomena leads to three main disadvantages: a decrease in utilization efficiency^18,19^, alternating /unstable dissolution of the anode and a low voltage caused by an IR drop across the layer of corrosion products, which is far away from theoretical Mg-air cell voltage of 3.1 V (1)

1 $$Mg+0.5{O}_{2}+{H}_{2}O\,\to Mg{(OH)}_{2}\,3.1V$$

Occurring theoretical limits to the anode potential in realistic scenarios due to the mentioned effects have been already discussed by Chen *et al*.^17^. However, a strategy to reach these limits in-service conditions has hitherto been lacking.

Several comparative studies aimed at finding effective corrosion inhibitors for Mg alloys have been performed^20–22^. However, little progress has been made in identifying optimal systems for Mg-air batteries. One of the reported approaches is based on the use of nitrate-based electrolytes instead of chloride-containing counterparts^23,24^. The non-ionic surfactant decyl glucoside has been recently shown to improve Mg-air battery performance by inhibiting anode self-corrosion^25^.

Recent work by Höche *et al*.^26^ has proposed an Fe-redeposition mechanism of Mg self-corrosion, which triggers a self-propagating process leading to strong microgalvanic corrosion and alkalinisation of the electrolyte and causing precipitation of Mg hydroxides on the metal surface. On the basis of this hypothesis, a new concept of Mg corrosion inhibition based on iron chelators has been reported^27^.

Using the chelator concept, we propose a non-trivial solution for controlling self-corrosion during the discharge of Mg anodes. We found that organic additives with dual functionality as strong Fe(III)- and mild Mg(II)-complexing agents significantly improved the performance of aqueous Mg-air batteries in terms of operating voltage, utilization efficiency and voltage stability.

**Figure 1:**
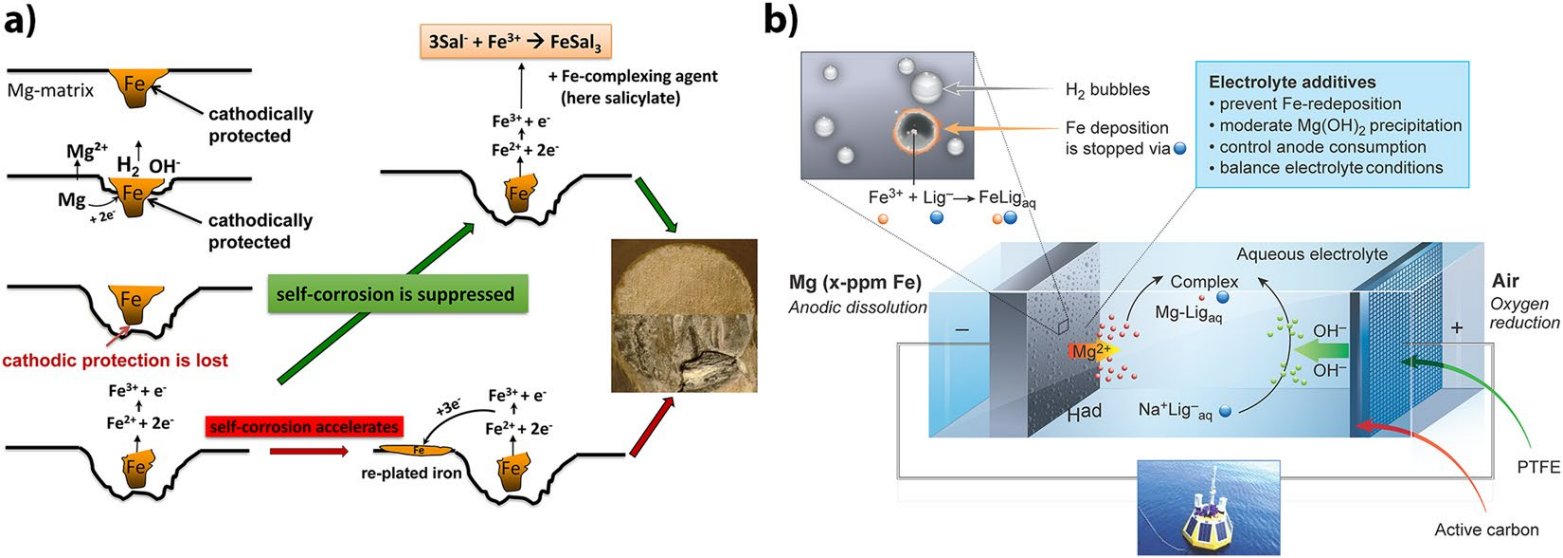
Additive working principles and mechanistic insights (**a**) Interaction of Fe impurities contained in the Mg anode with electrolyte additives (here salicylate, Sal) in aqueous Mg batteries. Re-plating of impurity particles accelerates self-corrosion of the anode. Interruption of the re-plating mechanism allows for the suppression of related anode fouling. **(b)** Principles of stabilization of aqueous Mg-air batteries by electrolyte additives, as illustrated in a synoptic summary (buoy, courtesy Martina Heineke, HZG). Main aspects of the additive effect are indicated and emphasize the scope of the discovery.

The working mechanism of the chelator additives (ligands), and recently introduced for being effective for Mg corrosion inhibition^27^, is illustrated in Fig. 1a. The strong cathodic reaction of water reduction on noble Fe at the corrosion potential of Mg induces anodic dissolution of Mg around the Fe-rich particles. After trenching of Fe is completed, cathodic protection is lost, and Fe undergoes rapid oxidation to form Fe^2+^/Fe^3+^ ions. These cations can be reduced back and plated onto the Mg surface, forming nanosized patches of pure iron. However, if the concentration of free Fe ions is greatly decreased by complexation with an organic ligand, self-corrosion of the anode can be inhibited, because the area of cathodic sites is either not growing or decreasing. Moreover, for effective chelating, the ligands and respective complexes with Fe must be electrochemically stable at the open-circuit potential of the Mg anode and at the potential of the discharging anode. The importance of this criteria was recently discussed by Hawthorne *et al*.^28^.

For battery applications (Fig. 1b), recent concept of corrosion inhibition^27^ must be further elaborated to account for the possible detrimental formation of Mg-containing precipitates. According to preliminary discharge tests in Half-cells^29^ and given the corrosion inhibition properties respectively the stability constants for complexes with Mg^2+^ and Fe^3+^, the following seven compounds were selected for testing: catechol-3,5-disulfonic acid disodium salt (Tiron), ethylenediaminetetraacetic acid dipotassium salt (K_2_-EDTA), and soldium salts of salicylate, glycolate, oxalate, nitrilotriacetate (NTA) and dodecylbenzenesulfonate (DBS).

## Results and Discussion

### Pre-testing of additives – hydrogen evolution as performance indicator

**Figure 2:**
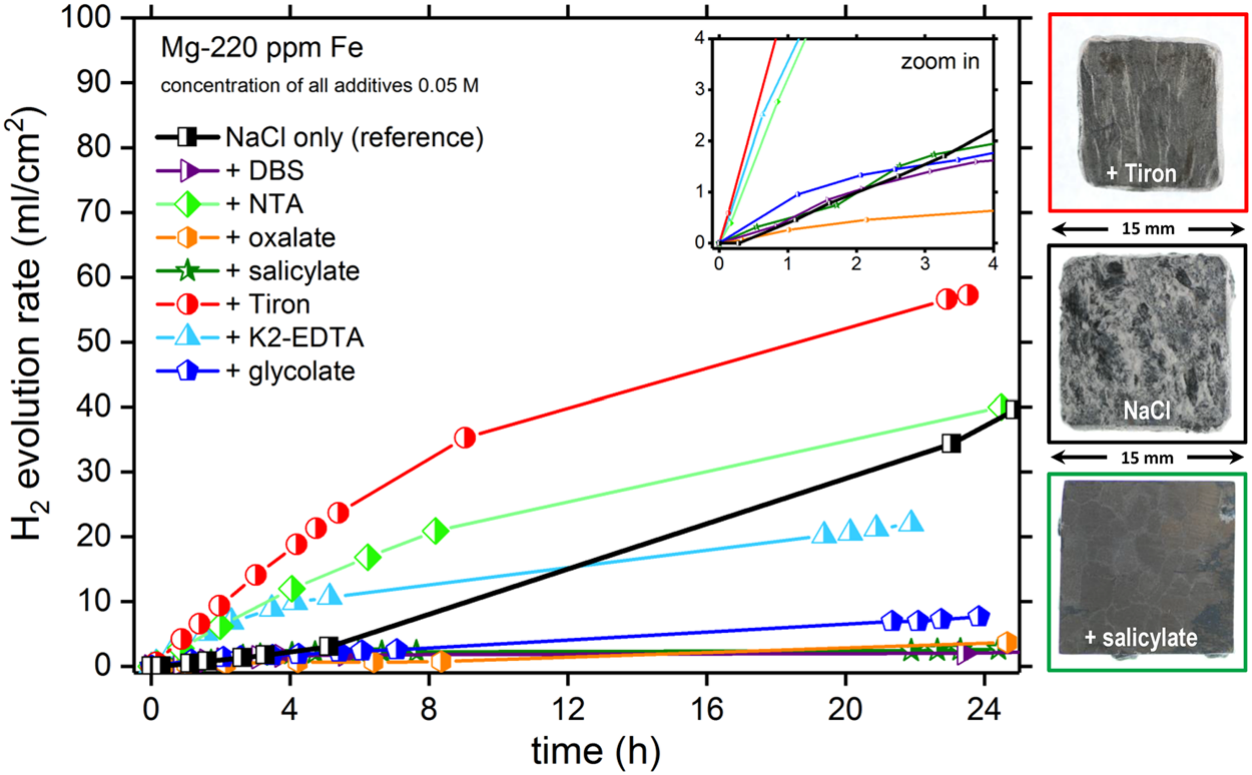
Monitoring anode self-corrosion via hydrogen evolution. Measured kinetics of Mg self-corrosion based on cumulative HER measurements by immersion tests in 0.5% NaCl solution and 0.5% NaCl solution containing different additives (0.05 M). Optical images (right) show the sample appearance after the tests with pure aqueous NaCl and aqueous NaCl modified with Tiron or salicylate.

The first parameter to be considered when evaluating the efficiency of electrolyte additives is the effect of the additives on the self-corrosion kinetics, because this effect directly correlates with the utilization efficiency, and influences the battery discharge voltage. One of the approaches to measuring the kinetics of Mg anode self-corrosion is based on the measurement of the evolution of cathodic hydrogen during immersion in a relevant electrolyte, because the extent of the HER directly correlates with the amount of dissolved Mg. Figure 2 shows the kinetics of hydrogen evolution measured for Mg self-corrosion at the open-circuit potential in the presence of either 0.5% NaCl without additives (reference) or NaCl with selected additives (0.05 M). The reference sample showed substantial HER that increased in rate over time, as is typical for a self-propagating process controlled by impurity redeposition. Additionally, in line with recent studies^26,30–32^, the sample was heavily corroded after 24 h of operation and was covered by Mg(OH)_2_ precipitates. This observation indicates that the Mg anode without adequate additive within the aqueous electrolyte is subject to strong self-corrosion even when the battery is in open-circuit conditions.

Initially (0–6 h), in the pure aqueous NaCl electrolyte, a low HER rate was observed followed by a steep increase of kinetics, because of the intensified cathodic reaction resulting from the increasing area of active Fe. In electrolytes containing Tiron, NTA or K_2_-EDTA, initial HER were even higher, in the order Tiron >NTA ≥ K_2_-EDTA.

However, the anode surfaces did not suffer from Mg(OH)_2_ precipitation like shown in the top-right of Fig. 2. Presumably, the measured enhancement of hydrogen evolution was related to the formation of highly soluble Mg complexes that shift the equilibrium toward Mg dissolution. The additives forced the removal of Mg^2+^ from the anode-electrolyte interface, thus leading to the formation of a fresh reactive surface, the exposure of new impurity inclusions, and the suppression of passive layer formation. Notably, although the size of all the anode samples was virtually the same at the beginning, the sample immersed in Tiron-containing electrolyte was consumed to a much greater extent than the one exposed to the NaCl electrolyte without additive (Fig. 2). The changing slope of the curves (Tiron, NTA, K_2_-EDTA) relates to the additive concentration decrease and pH increase^27^.

For solutions containing oxalate, glycolate, DBS or salicylate, the dissolution rate and HER remained low throughout the 24-hour period of data collection. Although the HER measurements in Fig. 2 were similar for these additives; the causes of such behaviour may be different. In the case of salicylate, this result can be explained by the stable [Fe^III^(salicylate)_3_] complex forming at high pH^33^, after a certain immersion time. Likewise, an alkaline pH is responsible for the change in dissolution kinetics in the case of DBS, but it causes the formation of Mg-DBS precipitates^20^ that are stable at high pH. Similar precipitation was observed after immersion in oxalate. In both cases, the precipitates blocked the anode surface (not displayed) and, as described below, decreased the battery performance in terms of discharge voltage due to an IR-drop across the precipitated layer of products. Mg dissolution rate in the presence of glycolate became stable at medium level of HER (constant slope after approx. three hours), probably because of a weaker Fe-chelating ability of the ligand.

All additives (except for DBS) formed complexes with both Fe^3+^ and Mg^2+^ ions, with varying stability constants *K*_i_, (Table 1). Salicylate complexes of Fe and Mg (log *K*_1_Mg^II^ = 4.7)^34^ were stable even at alkaline pH^35,36^. Glycolate formed weak complexes with Mg (log *K*_1_Mg^II^ = 0.92)^34,37^ and Fe-ions. EDTA formed highly stable Fe^38^ and Mg chelates (log *K*_1_Mg^II^ = 8.64)^39^. Oxalate^40^, similarly to DBS^20^, formed low-solubility Mg complexes that might have blocked the surface causing observed ohmic drop in Fig. 2, whereas NTA^41^ and Tiron^42^ complexes with Mg (both formed highly stable Fe-complexes) were soluble in aqueous electrolytes, thus representing a possible benefit. It should be noted that there is no general law directly linking dissolubility of Mg chelates with pH value. However, at certain pH Mg-ligand bonds can be broken an lead to precipitation of Mg(OH)_2_ from the complex.

**Table 1:** Half-cell testing related data and results.

complexing agent	stability constants			weight loss [mg] after 24 h of discharge test	utilization efficiency η [%]	pH after 24 h of discharge test (7 at 0 h)	discharge voltage [V]_Ag/AgCl_ after 24 h
	log K_x Fe_^III^	log K_x Mg_^II^	Ref.				
NaCl reference	—	—	—	20.2	13.5	10.2	−1.47
+Tiron	46.9 log K_3_	6.86 log K_1_	^34,46^	60.3	5.0	7.3	−1.75
+salicylate	36.8 log K_3_	4.7 log K_1_	^34^	10.0	27.2	9.6	−1.61
+K_2_-EDTA	24.23 log K_1_	8.64 log K_1_	^39^	34.9	7.8	7.2	−1.58
+glycolate	4.7 log K_i_	0.92 log K_1_	^34,37^	12.6	21.6	9.8	−1.47
+oxalate	20.2 log K_3_	4.38 log K_2_	^39^	12.5	21.8	9.9	−1.47
+NTA	24.32 log K_2_	10.2 log K_2_	^39^	41.7	6.5	8.3	−1.68
+DBS	n/a	*pK_0_^sp^ = 10.8	^47^	5.6	48.6	6.8	−1.38

Stability constants of complexes formed by the tested battery additives (0.05 M) and weight loss after the Half-cell discharge test in 0.5% NaCl solution (initial anode area = 0.5 cm²). Additionally, utilization efficiency η^18,19^, pH value and final voltage (errors within 3%) after 24 h discharge are shown. Substrate = commercial purity Mg (220 ppm Fe).

### Application of additives in test cells - discharge performance: Half-cell

**Figure 3:**
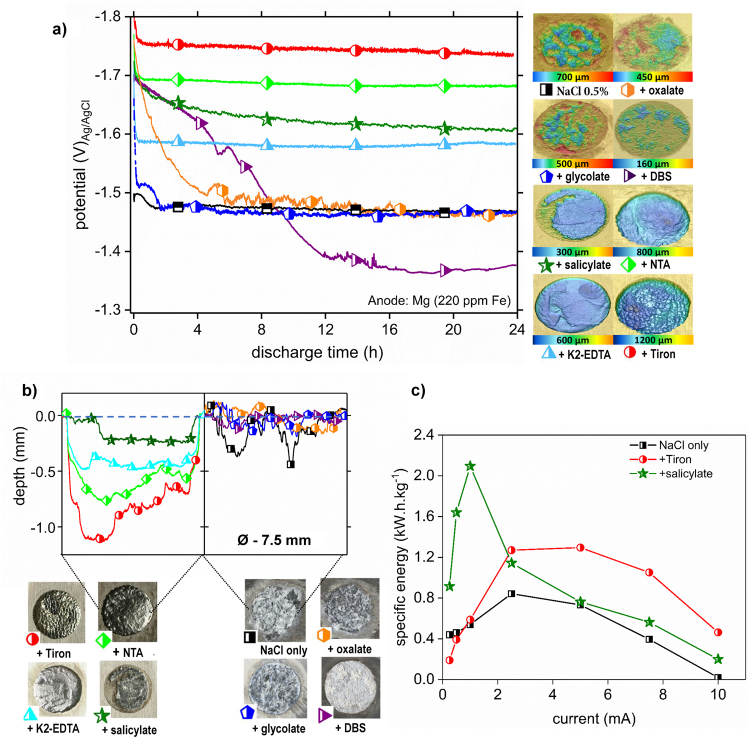
Half-cell tests - screening of additive performance. (**a**) Discharge curves obtained at 0.5 mA/cm^2^ constant current in the half-cell setup with 0.5% NaCl electrolyte and 0.5% NaCl containing additives (0.05 M). The anode was commercial purity Mg (220 ppm Fe). 3D maps (right) show the surface morphology of the anodes after the tests. (**b**) Optical micrographs (bottom) showing the surface appearance of the anodes after the Half-cell tests. Topological line scans (i.e. depth profiles, top) indicate the consumption of the anode material during the discharging shown in (**a**) after 24 h. (**c**) Specific Energy calculated for Mg (220 ppm Fe) discharged in electrolyte containing Tiron 0.05 M, Salicylate 0.05 M and without any additives at different applied currents.

In the next step, the proposed concept was tested in a Half-cell configuration using Pt counter electrode ensuring reliable and comparable test conditions for the anode discharge. Constant-current discharge curves for the selected additives are shown in Fig. 3a. The reference sample discharged in 0.5% NaCl at approximately −1.47 V_Ag/AgCl_ and showed a heavily attacked surface with localized corrosion and hydroxide precipitation (bottom-right Fig. 3b. Addition of oxalate and glycolate showed more negative discharge potentials vs. NaCl 0.5% reference at the beginning of the experiment, a result relating to the Fe complexing properties and the shift of the equilibrium at the anode surface towards Mg.

However, in both cases the potential became more positive over time, and was accompanied by the formation of insoluble products on the anode surface (Fig. 3b), probably because of the substantially lower stability constants of the Fe^3+^ and Mg^2+^ complexes (Table 1). After nine hours, DBS showed a more positive potential shift than the reference potential, owing to strong adsorption on the Mg surface followed by the precipitation of Mg-DBS and the related IR-drop through this formed layer (Fig. 3b). After the discharge tests in electrolytes containing salicylate and K_2_-EDTA, a moderate shift of the electrode potential was observed to persist at more than 110 mV more negative than that of the anode discharged in the pure aqueous NaCl electrolyte for 24 h. Figure 3b (bottom left) shows that no corrosion products were visible on the sample surfaces discharged in the presence of Tiron, NTA, K_2_-EDTA and salicylate. These chelating agents formed the strongest complexes with Mg^2+^ (Table 1) and correspondingly showed extended but relatively uniform anode consumption. The samples discharged in the presence of pure aqueous NaCl or NaCl with oxalate, glycolate or DBS salts (Fig. 3b), bottom-right), however, demonstrated minor anode consumption (depth profiles are masked by precipitated corrosion products). Despite the anode consumption the surfaces of the Mg anodes were brightly shining after the discharge tests and were found to have granular microstructures in the cases of adding NTA Fig. 3b and Tiron (shown in Figs 2 and 3b, which also demonstrated the most negative discharge potentials.

However, these more negative potentials (high operating voltages) were accompanied by high anode consumption and low utilization efficiency (Table 1), as assessed by weight loss measurements after the corrosion products were removed. The rate of anode consumption and related weight loss was proportional to the stability of the Mg complex with the additive ligands (Table 1), whereas the cell voltage correlated with the Fe complex stability. The measured values of utilization efficiency (η = theoretical weight loss/measured weight loss) are lower than previously reported values^18,19^. This difference is related to the significantly lower chloride content^43^ used in the tests and also to the high amount of impurities, thus making the used anodes much more prone to self-corrosion than alloys used elsewhere^18,19^. Remarkable, that utilization efficiency was more than twice higher in salicylate containing electrolyte compared to the reference.

A comparison of the weight loss data and the discharge potentials revealed two additive selection strategies based on the specific application: a longer discharge time (durability) could be obtained by using either K_2_-EDTA or salicylate if mild operating voltages are sufficient (110–140 mV above reference) or higher voltage (additionally 210–280 mV) could be achieved at the expense of faster anode consumption by using either NTA or Tiron. These tunable properties enable the possibility of tailored discharge. The utilization efficiency for the most balanced additive salicylate (compromise voltage/durability), was in the range of 30%, a value twice as high as that of the NaCl electrolyte with no additives. Along with a 140 mV higher operating voltage than using the pure aqueous NaCl electrolyte, this result represents a considerable improvement.

For both strategies, represented here by salicylate and Tiron respectively, the performance boost was complementary monitored via specific energy measurements as shown in Fig. 3c. The results show a current dependency which originates from the contribution of self-corrosion related current. At low discharge currents it is stronger and the working mechanism of salicylate takes control. On the contrary when the current is increased, Tiron, which stimulates dissolution of Mg, triggers the performance. It is assumed that the strength of the enhancement thereby strongly relates to the surface constitution, its polarization properties and the occurring IR drop (film and/or electrolyte).

### Application of additives in test cells - discharge performance: Full-cell (Mg-air)

**Figure 4:**
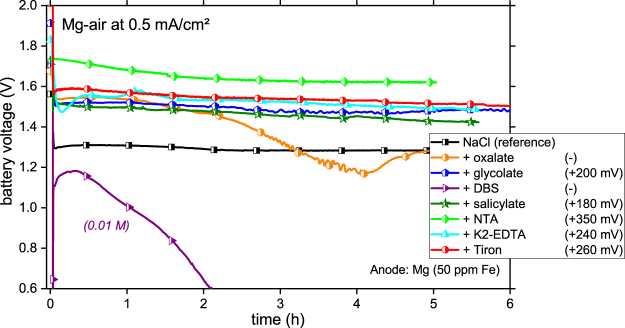
Application of additives in a full-cell scenario. Comparison of Mg-air battery (carbon fabric as the cathode) discharge performance measured at 0.5 mA.cm^−2^ in 0.1 M NaCl electrolyte containing complexing agents (concentration 0.01 M for DBS and 0.05 M for other additives). The anode was high purity Mg (50 ppm Fe). The average battery voltage increase vs. the reference is listed.

Half-cell tests have demonstrated that addition of complexants can significantly affect kinetics of discharge and self-corrosion of Mg-based anodes. However, such tests exclude the effect of cathode material (here carbon fabrics) and its kinetics (here oxygen reduction) on the cell and anode discharge. Thus, only Full-cell tests can validate the additive effect in end-use batteries. Respective Full-cell discharge curves of the Mg-air battery in the electrolytes with different complexing agent additives are shown in Fig. 4.

Despite different impurity grade of anode material the curves of most additives follow the trend obtained by Half-cell tests. Good performance in terms of battery voltage for cells containing NTA and Tiron was demonstrated; the slightly weaker performance of Tiron probably relates to the lower Fe impurity content of 50 ppm in the Full-cell test. The better (than in the Half-cell) performance of the cell containing glycolate was probably observed because the limited stability of the Mg-glycolate complexes, owing to the high pH in the Half-cell, was sufficient during Full-cell testing. K2-EDTA and salicylate performed stable at higher cell voltage. The expected detrimental effect due to insoluble complex formation and adsorption was explicitly shown for DBS and oxalate.

### Correlation and summary of findings

The outcome of all tests is summarized in Table 2. For Mg anodes, the self-corrosion and fouling of the electrode should be controlled to increase the discharge voltage and enhance the utilization efficiency using e.g. NTA, Tiron, K2-EDTA or salicylate additive, whereby anode consumption must be moderated to retain the advantage of volumetric capacity. Thus, for battery applications, the additives must meet the following requirements:

1. The additive must be able to form a highly stable iron complex (Fe–ligand) like shown for Tiron or salicylate via discharge tests.
2. The ligands and respective complexes with Fe must be stable at the open-circuit potential of the Mg anode and at the potential of the discharging anode (e.g., to prevent electro-(back)-deposition (analog^28^); it was not observed for all additives).
3. The additive must be able to form soluble complexes with Mg^2+^ (Mg–ligand) to prevent detrimental formation of precipitates on the anode surface like shown in Fig. 3b. The stability of such complexes must be moderate (e.g. like salicylate) to avoid enhanced dissolution of Mg by shifting the chemical equilibrium towards the accelerated formation of soluble complexes (e.g. like NTA and Tiron).

**Table 2:** Physicochemical processes vs. battery performance parameters.

acting process	formation of			fluctuating dissolution	adsorption	convection/flow	comment
controlled property	Fe–ligand	Mg–ligand	insoluble reaction products				
utilization efficiency							max. improvement in this work: factor 2
voltage							theoretical anode limit: −2.1 [V]_Ag/AgCl_^[1]^
Example 1: Tiron	strong	strong	no	no	no	weak effect	high voltage but low durability
Example 2: oxalate	moderate	moderate	yes	weak	yes	effect	battery fails as result of anode blockage
Example 3: salicylate	strong	moderate	weak	no	weak	weak effect	moderate voltage with high durability

Control of the discharge behaviour by physical/chemical interaction processes (increase, decrease or no change) and respective additive examples.

Formation of Mg complexes is expected to have a two-fold effect that must be balanced (voltage vs. durability). Its positive influence arises from a) preventing the formation of Mg(OH)_2_ that otherwise blocks the Mg surface, and b) providing local stable steady-state conditions to maintain Mg dissolution. The negative effects of the formation of Mg complexes are a) the sparingly soluble Mg complexes, which (despite being effective for corrosion inhibition) are detrimental for the discharge voltage, owing to anode blockage, and b) strong Mg complexes accelerate the dissolution of Mg and parasitic anode consumption.

The desired balance between the stability of the complexes with Fe and Mg can be achieved by the proper selection of complexing agents that form targeted complexes with both cations, or by combining different complexing agents in the same electrolyte. In addition, the optimization of the additive concentration is likely to result in considerable improvement of the voltage and utilization efficiency.

For tailoring the battery discharge, adjustment of the interaction processes as shown in Table 2 is required. The additives offer the respective toolbox (three examples in). Convection (e.g. in flow cells) is named to complete the list since it directly affects cell pH and the adsorption kinetics. Independent from Table 2 the additive concentration within the electrolyte determines the dissolution of the anode as well. Considering the mentioned aspects, adapting the electrolyte reservoir dimension and the ageing of the electrolyte itself are mandatory. Consequently, synergistic additive mixtures in combination with a systematic electrolyte design, including the complementary adjustment of battery components, must be applied to unleash the full potential of this technology sleeping couple of decades. Salicylate (Fig. 2) and K_2_-EDTA efficiently block anode self-corrosion and consume anode at moderate rate. Both could be applied to extend the life-time of e.g. rescue-batteries based on Mg. In contrast, NTA and Tiron (Fig. 2), which form soluble complexes, offer adequate Mg dissolution conditions and a low anodic polarization. Unfortunately, named benefits come at the price of anode consumption and decreased utilization efficiency. They are a good option if a high and stable voltage is required for short periods.

The scientific value of this work becomes evident by comparing the chelator strategy to other approaches based on alloying^11,44^, microstructural optimization^45^ or battery (component) design^8^. The proposed idea has been shown to be very promising since it allows effective control of anode activity very close to the theoretical limit thereby avoiding use of hazardous alloying elements. Additionally, the admixture of respective chemicals is economical attractive, its technical handling is simple and most of them are environmentally benign.

## Conclusion

New strategy on improvement of Mg-electrode discharge characteristics via addition of iron complexing agents is reported for the first time. The electrolyte additives targeting tailored discharge of primary magnesium-air batteries must fulfil three important requirements.

1. The first is an adequate stability of the resulting Fe complex at neutral and alkaline conditions.
2. Second, the complexing agents must be stable under the occurring local (polarized) conditions, to retain Fe-ions during the discharge process.
3. Third, the Mg-additive complexes should be soluble to prevent anode fouling.

Further technical optimization of the additives is required to identify the compounds with the most favourable log *K*_Mg_ and log *K*_Fe_. The best system tested in this work was salicylate, which yielded a utilization efficiency twice that of the reference NaCl at 140–180 mV of higher discharge potential. An even better potential (210–350 mV vs. reference) could be achieved with Tiron or NTA but at the expense of faster anode consumption. Likewise, additive mixtures and optimal concentrations should be assessed to achieve the highest possible discharge potential and utilization efficiency. Further progress requires research not only on the interaction of the additives with novel air cathodes (e.g. activated carbon), but also towards a cell design that optimizes the additive interaction environment (pH etc.). To the end of adapting this technology towards tailored discharging and enhanced performance, clear requirements have been established for the electrolyte additives and its application towards a renaissance of primary Mg batteries.

## Methods

### Chemicals

The following chemicals were used as additives: sodium salicylate (cat. no. 71945, Sigma-Aldrich); glycolic acid (cat. no. 124737, Sigma-Aldrich), dodecylbenzenesulfonic acid sodium salt (DBS, cat. no. 289957, Sigma-Aldrich), 4,5-dihydroxy-1,3-benzenedisulfonic acid disodium salt monohydrate (Tiron, cat. no. 89460, Sigma-Aldrich); nitrilotriacetic acid disodium salt (NTA, cat. no. N0128, Sigma-Aldrich), ethylenediaminetetraacetic acid dipotassium salt dehydrate (K2-EDTA, cat. no. 819040, Merck) and oxalic acid dihydrate (cat. no. 006053, Chempure). The pH of 0.05 M solutions of the complexing agents was adjusted by adding NaOH to reach a final value in the range of 6.7 to 7.2.

### Anode materials

The anode Mg materials (CP grade) were produced in the HZG castshop. The Mg impurity content was measured by spark discharge-optical emission spectroscopy (SD-OES) with spark analyser vision software (SPECTROLAB). The bare material was cut into pieces (16 mm × 16 mm × 4 mm resp. 28 mm × 43 mm × 2.5 mm), ground, polished and rinsed with ethanol.

### HER-testing

Hydrogen evolution tests were performed using eudiometers (cat. no. 2591–10–500 from Neubert-Glas, Germany). The immersion solution was 0.5% (0.085 M) aqueous NaCl with or without a complexing agent/battery additive.

### Microscopy

Optical microscopy was performed using Leica DMI500 system. 3D maps were produced using a Keyence VK-9700 confocal laser microscope.

### Cell testing

The discharge tests were performed using commercial purity Mg containing 220 ppm Fe in a Half-cell with a Pt cathode connected via a salt bridge (Figure S1). In this setup, the influence of rapid pH change due to the cathodic reaction was moderated. Discharging (in Half-cell mode) was performed using an Interface 1000 system (Gamry) with a three-electrode setup in a 330 mL cell with Pt as the counter electrode and Ag/AgCl_sat_ as the reference electrode. Weight loss was determined by measuring the weight difference of the samples before and after the discharging tests after removal of deposits by etching with chromic acid, cleaning and drying. The size of the samples was 16 mm × 16 mm × 4 mm.

The additives were also tested in primary Mg-air battery (flow-cell setup (Figure S2)) by using high-purity Mg containing 50 ppm Fe (already commercial available). The cells were discharged with a constant current density of 0.5 mA cm^−2^ in 0.1 M NaCl. Plates of the high-purity material with dimensions of 28 mm × 43 mm × 2.5 mm were used as the anode material, and an activated carbon fabric (Kynol Europa GmbH) with a gas-diffusion layer of PTFE was used as the cathode material. The electrodes were separated at a distance of 1 cm, and the cell (1 L) was filled with the electrolyte.

### Specific Energy

Half-cell discharge tests with respect to the quantification of the specific energy were carried out at different currents (ranging from 0.025 mA to 10 mA) with the electrolyte containing Tiron, sodium salicylate and electrolyte without any additives (NaCl 0.5%). Specific energy obtained from 24 hours of anode discharge was calculated by the following formula:

2 $$specific\,energy=\,\frac{V.I.t}{w}[\frac{Wh}{kg}]$$

where V is the average voltage obtained from discharge curve; I is the applied current; t is the time of discharge; and w is mass loss of anode after discharge.

Note that for the cases of reference electrolyte (NaCl 0.5%) at currents 10 mA and 7.5 mA, and for the electrolyte containing sodium salicylate at current of 10 mA, the battery fails (here, battery failure is when voltage becomes positive) before reaching the 24 hours of discharge. Discharging was stopped at failure point and all variables were calculated and measured corresponding to that point.

Hydrogen evolution, discharge and corresponding weight loss measurements were performed at least twice for all the chelating additives. The results correlated within 5%.

Each specific energy value was obtained from at least three discharge tests at corresponding applied current and electrolyte.

### Data availability

The datasets generated during and/or analyzed during the current study are available from the corresponding author on reasonable request.

## Electronic supplementary material

supporting information

Battery Boost with Salicylate
