# Supplementary material for: Performance boost for primary magnesium cells using iron complexing agents as electrolyte additives
Source: Sci Rep. 2018 May 15;8:7578. doi: 10.1038/s41598-018-25789-8 (PMC5953936; doi:10.1038/s41598-018-25789-8)
Supplement: Supplementary file 1 — supporting information [file 41598_2018_25789_MOESM1_ESM.pdf]

Supporting Information

Performance boost for primary magnesium cells using iron complexing agents as electrolyte additives

Daniel Höche*[,1,2], Sviatlana V. Lamaka[1], Bahram Vaghefinazari[1], Tobias Braun[3], Rokas P. Petrauskas[4], Maximilian Fichtner[3,5], Mikhail L. Zheludkevich[1,6]

[1] Helmholtz-Zentrum Geesthacht (HZG), MagIC-Magnesium Innovation Centre, Max-Planck Str. 1, 21502 Geesthacht, Germany.
[*] daniel.hoeche@hzg.de
[2] Helmut-Schmidt-University University of the Federal Armed Forces, Faculty of Mechanical Engineering, Holstenhofweg 85, 22043 Hamburg, Germany.
[3] Helmholtz Institute Ulm (HIU), Helmholtzstr. 11, 89081 Ulm, Germany.
[4] University of Vilnius, Department of Inorganic Chemistry, 03225 Vilnius, Lithuania.
[5] Karlsruhe Institute of Technology (KIT), Institute of Nanotechnology, Hermann-von-Helmholtz Platz 1, 76344 Eggenstein-Leopoldshafen, Germany.
[6] University of Kiel, Faculty of Engineering, Kaiserstrasse 2, 24143 Kiel, Germany.

Supplementary Text

Battery safety, inflammability and other hazard properties are key aspects of modern battery developments. In this context, the shown future-oriented Mg-air-cells (incl. additives) have outstanding property portfolio. Additionally, they do not have any packaging restrictions, are environmentally benign and are partially recyclable (replacement of anode).
Remark: Only the use of NTA should be restricted because, although its toxicity is low, it is considered to be potentially carcinogenic to humans.

Figure S1. Half-cell setup for discharge tests. where RE is the reference electrode, WE is the working electrode, CE is the counter electrode and SB is the salt bridge. All electrodes are connected to Gamry Interface 1000 potentiostat and measurements are carried in standard three electrode configuration.

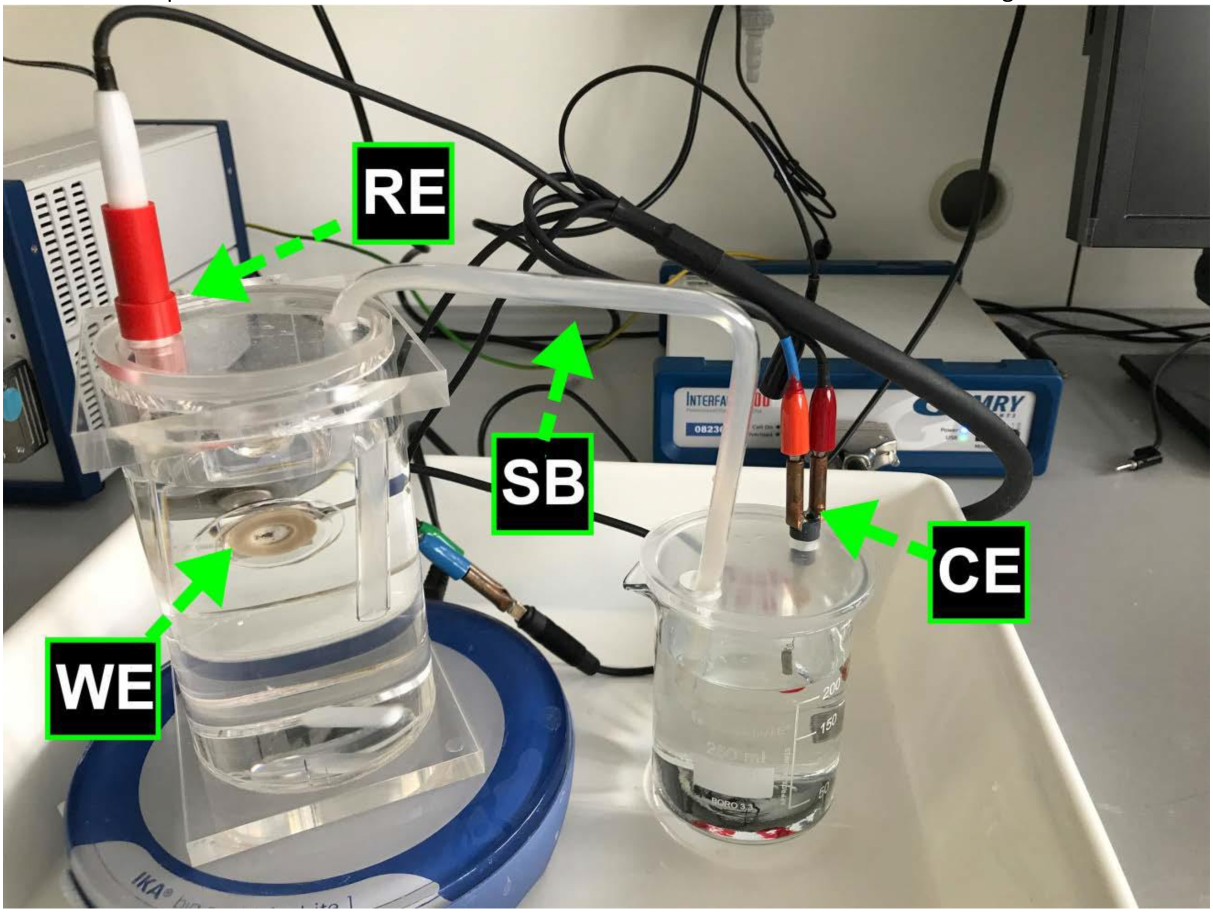

Figure S2. Flow cell setup used for Full-cell testing. The flow cell setup contains three main parts: an anode casing (Part 1), electrolyte casing (Part 2) and a cathode casing (Part 3). The anode (30 mm × 60 mm × 2.5 mm) is pressed on part 2). The electrolyte flows through a compartment of 28 mm × 50 mm × 10 mm with a flow rate of 6-10 mL min^{-1}. The cathode casing comprises holes for oxygen exchange from ambient air and a compartment for the air cathode (30 mm × 60 mm× 2.5 mm) which is pressed on part 2). The three parts are assembled as shown on the right image and tightened with screws. The anode and cathode are fixed with electrical connectors (screws) on the rubber of the electrolyte casing.

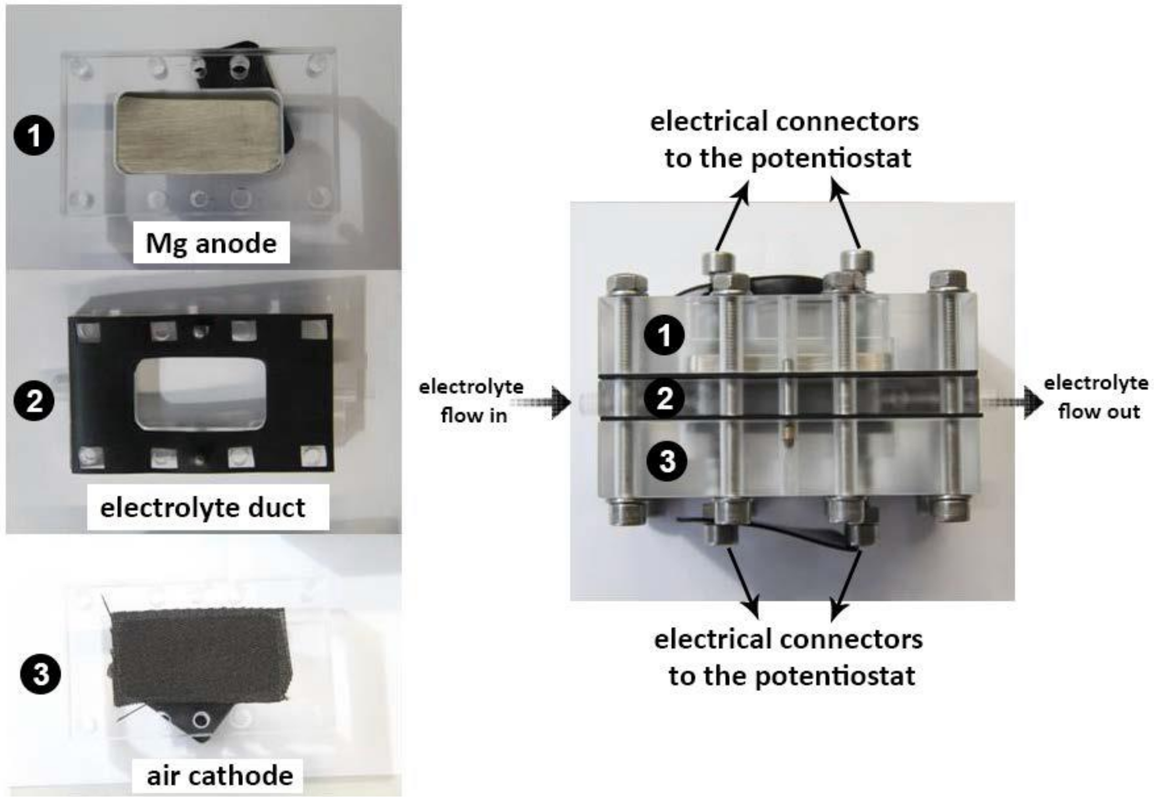

Table S1. Elemental composition of commercial purity magnesium anodes according to optical emission spectroscopy (OES) measurements.

	Element	**Al**	**Ca**	**Ce**	**Cu**	**Fe**	**La**	**Mn**	**Ni**	**Si**	**Zn**	**Zr**	**Mg**
anode used for **half-cell** setup	Impurity level, ppm	130 ±15	4 ±3	<4	5 ±3	**220** ±30	<5	150 ±65	<2	53 ±45	4 ±2	5 ±4	99,94%
anode used for **full-cell** setup	Impurity level, ppm	50 ±6	<1	<4	<1	**51** ±30	<5	8 ±4	<2	<1	10 ±5	5 ±4	99,98%

Movie S1. (provided in a separate file)

The video represents a full battery test by a magnesium-air prototype battery. A block of commercially pure magnesium (with 50 ppm Fe impurity) was used as anode. Activated carbon (catalyst: manganese oxide) as air cathode was supplied from Gaskatel in Germany (incl. PTFE gas diffusion layer). The battery was discharged with electrical load of an LED in the circuit, while a multimeter was measuring the voltage delivered to the LED.

A very rapid raise in delivered voltage at the beginning of discharge was observed, followed by a gradual decrease over discharge time. Magnesium anode failed after 20 hours of discharge when electrolyte was 0.5% NaCl reference without any additive. Failure criteria was set when the delivered voltage is not able to light the LED (threshold voltage is about 1.5 V). After 20 hours of discharge the anode surface was completely black (iron enriched) and the electrolyte lost its transparency due to the fouling by magnesium corrosion products.

However, addition of sodium salicylate at the concentration of 0.05M to the electrolyte led to remarkable boost in performance of battery. Sodium salicylate was able to keep the delivered voltage to values higher than 1.64 V for more than 14 days. Anode surface was almost free of black regions and a visible corrosion product layer which would cause IR drop was lacking. Additionally, the electrolyte remained transparent with a light yellowish shade after 14 days of discharge.
